# Repeated Synchronous Aspiration and Ingestion of a Sharp Metallic Foreign Body in a Mentally Disabled Adolescent

**DOI:** 10.1155/2019/1296061

**Published:** 2019-01-23

**Authors:** Dragan Subotic, Nikola Atanasijadis, Dejan Moskovljevic, Dragana Asujic

**Affiliations:** ^1^Clinic of Thoracic Surgery, University Hospital Basel, CH-4031, Switzerland; ^2^Clinic of Thoracic Surgery, Clinical Center of Serbia, 11000 Belgrade, Serbia; ^3^Clinic of Anesthesiology, Clinical Center of Serbia, 11000 Belgrade, Serbia

## Abstract

In a mentally disabled adolescent, bronchoscopic extraction failure of a metallic foreign body from the left main bronchus was followed by mediastinal emphysema. At thoracotomy, a part of the metallic hook was found to protrude through the main bronchus, just by the descending aorta. The foreign body was removed and the bronchus sutured. After the thoracotomy closure, laparotomy was performed with removal of metallic pieces from the stomach. After three years, a repeated metallic foreign body aspiration as confirmed by the chest radiography ensued, with metallic pieces in the bowels as well. With the surgical team on site, rigid bronchoscopy was done and the foreign body extracted from the intermediate bronchus. Metallic pieces left the digestive tract spontaneously after a few days. In conclusion, the appropriate preoperative workup and timing for surgery are essential for the treatment outcome of this life-threatening condition; because of the high likelihood of the major airway injury, such procedures should be done with a surgical team available whenever possible.

## 1. Introduction

The role of fiber-optic bronchoscopy in the treatment of foreign body (FB) has been well established [1–3]. Till the end of the 20th century, the rigid bronchoscopy used to be reported as a possible option either as upfront intervention or in cases where flexible bronchoscopy failed, especially in the case of sharp FBs, usually embedded in the scar tissue [4]. The optimal approach for the FB aspiration in mentally disabled persons, especially in the case of repeated aspiration, has not been sufficiently evidence based.

## 2. Case Report

In a 19-year-old, mentally disabled male, chest radiography was done because of a sudden episode of cough. Metallic, hook-shaped foreign bodies were identified in both the main bronchi.

**Figure 1:**
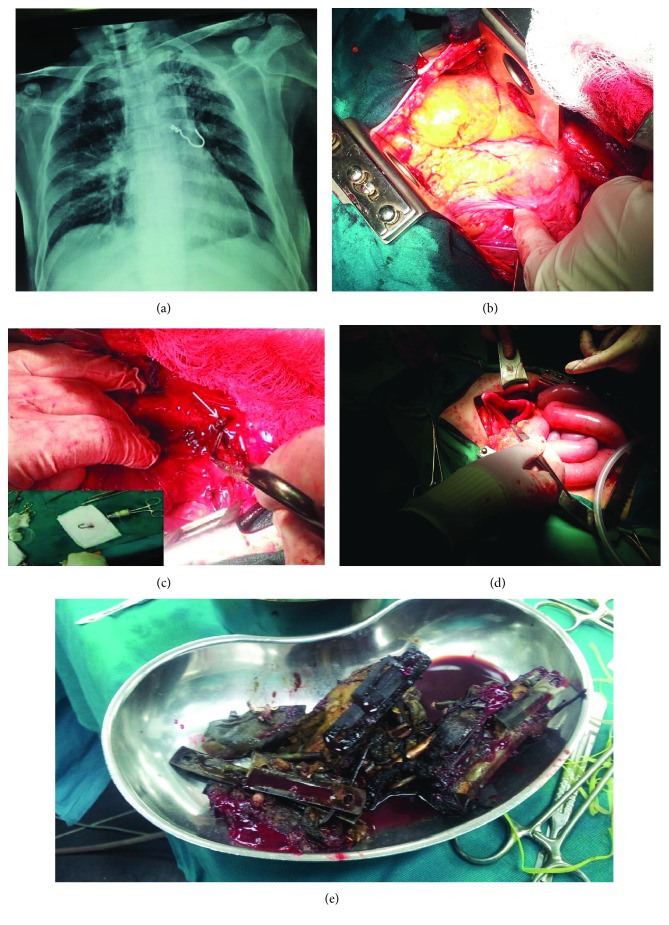
The first episode of FB aspiration and ingestion. (a) PA radiography on admission. (b) Mediastinal emphysema at thoracotomy. (c) FB (arrow) protruding through the left main bronchus (insert: FB removed). (d) Laparotomy and gastrotomy. (e) Metallic FBs removed from the stomach.

The right-sided FB was removed by fiber-optic bronchoscopy in the regional hospital, whilst the left-sided extraction failed with the left-sided FB persisting in the left main bronchus (Figure 1(a)).

Upon urgent admission in a tertiary institution, extraction was attempted by rigid bronchoscope under general anesthesia. Bronchoscopic extraction failed, associated with some bleeding and subcutaneous emphysema immediately after the intervention. The increasing mediastinal and subcutaneous emphysema raised suspicion about an iatrogenic airway lesion, so surgery was indicated. Esophageal injury was previously ruled out by esophagoscopy, revealing many metallic FBs in the stomach. At thoracotomy, a significant mediastinal emphysema (Figure 1(b)), together with diffuse adhesions, was noticed. After the lung liberation, a proximal 0.5 cm of the noncurved part of the metallic hook was found to protrude through the perforated membranous wall of the left main bronchus, 1 cm away from the descending aorta (Figure 1(c), arrow). The part of the FB protruding outside the bronchus was grasped by the clamp and, by following the curved shape of the FB, gentle maneuvers were applied by pulling the sharp end (hook) of the FB in the direction outside the bronchus. The FB was removed from the bronchus (insert on Figure 1(c)) without the need for additional bronchotomy. The remaining 10 × 1 mm defect in the bronchial wall, caused both by manipulations during a bronchoscopic extraction attempt and subsequent surgical extraction, was sutured by interrupted PDS 3-0 stitches, and the lung fully inflated. No air leaks appeared during the water test. Having in mind the dimensions of the defect and tensionless suture, no suture-line protection was performed.

After the thoracotomy closure, laparotomy was done and several sharp metallic pieces of different shapes were removed from the stomach (Figures 1(d) and 1(e)). This was followed by an uneventful postoperative course and the discharge after 14 days.

**Figure 2:**
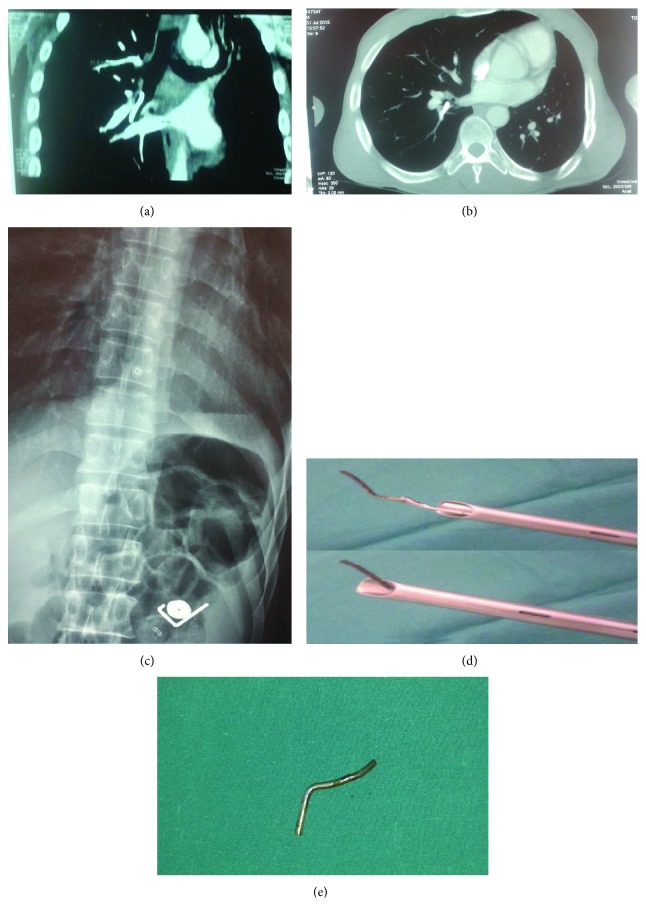
Repeated FB aspiration and ingestion. (a,b) CT of the thorax on admission. (c) FBs in the abdomen. (d) Illustration of the bronchoscopic maneuvers. (e) Metallic FB removed.

After three years, the patient was urgently readmitted for the new episode of the metallic foreign body aspiration (Figures 2(a) and 2(b)). The abdominal radiography revealed metallic pieces in the digestive tract as well (Figure 1(c)). With the surgical team on site, rigid bronchoscopy under general anesthesia was done. As the tip of the FB was not impacted in the mucosa, it was possible to grasp it with the rigid biopsy forceps and to withdraw it up to the tip of the bronchoscope. Because of the curved shape of the FB and the impossibility to remove it through the instrument, the FB and bronchoscope were pulled out from the patient together, with the FB firmly grasped, followed by reintubation with the same bronchoscope (Figures 2(d) and 2(e)). After a careful check-up for bleeding and mucosal damage, the patient was extubated.

Metallic pieces left the digestive tract spontaneously after a couple of days.

## 3. Discussion

In this patient, suspicion about FB aspiration was raised owing to sudden cough, similar to other reports on mentally retarded patients [5].

The key point concerning the two instances, separated by a 3-year interval, is the difference in treatment priorities.

In the first instance, the need for surgery was obvious, having in mind a progressively developing mediastinal emphysema, unavoidable mediastinitis within the following 24-48 hrs and high probability of the lethal outcome if left untreated. Here, the first pitfall related to the preoperative workup: in this patient, esophagoscopy was done before thoracotomy, with the aim to rule out iatrogenic esophageal injury. But additional metal pieces were discovered in the stomach only owing to an experienced endoscopist, who extended the exploration more distally, as usually is the case of repeated swallowing of foreign bodies in psychiatric patients [6]. Otherwise, the metal in the stomach would have been overlooked (Figure 1(a)).

The second lesson of the first instance is very instructive: unless the sharp, curved part of the sharp metallic hook is clearly visible within the bronchus, as it was not the case, bronchoscopic extraction can be cautiously attempted by applying some maneuvers as previously described [7]. However, it should not be repeated in case of failure because of the high likelihood of the major airway injury.

The next lesson relates to the timing of surgery: could it have been postponed for at least 24-48 hrs, to start with antibiotics or to buy some time for hemoptysis to stop? It is clear that the risk for mediastinitis was high enough to eliminate such a scenario, but the answer to this question was additionally clarified during the operation (Figure 1(c))—the longer part of the metallic hook was in close contact with the descending aorta. As the great part of the pleural space was obliterated by adhesions, even the lung manipulation during the lung liberation could cause additional FB displacement towards the aorta.

Both in the first and second situations, the usefulness of the computerized tomography (CT) of the thorax could be discussed. Its role is well established in the case of suspected or unsuspected aspiration of radiolucent foreign body [8]. If the foreign body is entirely endobronchial, coronal and sagittal projections are of help in planning bronchoscopic removal, as it was the case before the second intervention.

The only uncertainty in the case of thin, curved metallic devices, as in the repeated aspiration, could be the choice between flexible and rigid bronchoscopy. The choice of the rigid bronchoscopy in the current report was determined by the dimensions and shape of the metallic FB, ruling out the possibility to remove it through the bronchoscope. Grasping the FB, pulling it out together with the instrument and reintubation of the patient, was the only possible option in the patient. In this situation, even the combination of the endotracheal tube and flexible bronchoscopy, as previously reported [7], should not contribute to the safety of the procedure, as compared to the selected method.

Related to possible failure of bronchoscopic removal, we agree that such a procedure should be done in the operating room whenever possible and with a patient completely prepared for surgery, as previously suggested [9].

## 4. Conclusion

In mentally disabled patients, rigid bronchoscopy can be an appropriate procedure for the sharp metallic FB extraction. In addition to the skilled and well-equipped endoscopical team with support of surgery whenever possible, the overall approach should be carefully planned and adjusted to the particular situation.
